# Using Integrative Behavior Model to Predict COVID-19 Vaccination Intention among Health Care Workers in Indonesia: A Nationwide Survey

**DOI:** 10.3390/vaccines10050719

**Published:** 2022-05-04

**Authors:** Sukamto Koesnoe, Tommy Hariman Siddiq, Dicky C. Pelupessy, Evy Yunihastuti, Ghina Shabrina Awanis, Alvina Widhani, Teguh Harjono Karjadi, Suzy Maria, Anshari Saifuddin Hasibuan, Iris Rengganis, Samsuridjal Djauzi

**Affiliations:** 1 Allergy and Clinical Immunology Division, Department of Internal Medicine, Faculty of Medicine, Universitas Indonesia/Cipto Mangunkusumo Hospital, Jakarta 10430, Indonesia; evy.yunihastuti@gmail.com (E.Y.); ghishabrina@gmail.com (G.S.A.); alvina.widhani@gmail.com (A.W.); tghsemar59@gmail.com (T.H.K.); suzyduri@gmail.com (S.M.); sorihsb@gmail.com (A.S.H.); irisrengganis@yahoo.com (I.R.); samsuridjal@yahoo.com (S.D.); 2 Faculty of Psychology and Education, Universitas Al-Azhar Indonesia, Jakarta 12110, Indonesia; tommy@ujisdm.com; 3 Faculty of Psychology and Education, Universitas Indonesia, Depok 16424, Indonesia; dickypsy@ui.ac.id

**Keywords:** health behavior, COVID-19 vaccines, intention, acceptance, health care workers

## Abstract

*Background*: Health care workers (HCWs) are a high-priority group for COVID-19 vaccination for several reasons. Health behavior theory-based studies on the intention or acceptability of COVID-19 vaccination among Indonesian HCWs is lacking. Using an integrated behavioral model, this research sought to identify Indonesian health care workers’ intentions to obtain COVID-19 vaccines. *Methods*: A countrywide cross-sectional questionnaire-based survey was conducted. The questionnaire was constructed on the basis of IBM (integrated behavioral model) constructs and scored on a seven-point bipolar scale. A hierarchical multivariable regression was used to evaluate the fit of the predictor model as well as the correlations between variables in the study. *Results*: 3304 people responded to the survey. A model combining demographic and IBM characteristics predicted 42.5 percent (adjusted *R*^2^ = 0.42) of the COVID-19 vaccination intention. Vaccination intention was associated with favorable vaccine attitudes, perceived norms, and self-efficacy. Among the determining constructs, behavior belief predicted vaccination intention the best. Being female, being married, having a history of COVID-19 infection, living outside Java Island, and having a low income were all linked to lower vaccination intentions. *Conclusions*: This study confirms the IBM model’s robustness in predicting health care workers’ intention to vaccinate against COVID-19.

## 1. Introduction

Despite the fact that the continuing coronavirus disease 2019 (COVID-19) pandemic has caused global devastation, COVID-19 infections presently lack a definitive or viable cure. As a result, the only way to end this pandemic is with COVID-19 vaccinations. The development of COVID-19 vaccines started in 2020 [1]. The WHO Strategic Advisory Group of Experts on Immunization (SAGE) roadmap prioritizes vaccination of health care professionals; those at high risk of mortality or severe sickness; and people at high risk of SARS-CoV-2 infection due to lack of social distance, such public servants [2].

Health care workers (HCWs) are a high-priority demographic for COVID-19 immunization for a variety of reasons. First and foremost, they are vulnerable to COVID-19 infections. According to LaporCOVID-19, an independent data initiative organization, 2066 Indonesian health care professionals have perished as of January 18th, 2022 [3]. Moreover, a meta-analysis found that around 15.6 percent of patients with COVID-19 infections seen by health care personnel were asymptomatic [4]. HCWs with asymptomatic COVID-19 infections may become a source of transmission to patients, families, and coworkers, especially to older adults and to those with chronic conditions at increased risk of fatality due to COVID-19 infections [5,6].

When COVID-19 vaccination becomes widely accessible, one of the challenges will be public acceptance, particularly among priority health care personnel. Previous research has revealed a range of attitudes toward or acceptability of COVID-19 immunization among health care personnel. According to a survey conducted in Canada, 80.9 percent of health care personnel have embraced vaccination [7]. Another Saudi Arabian study found that only 64.9% of health care personnel surveyed were willing to receive vaccinations [8]. Harapan et al. stated that 95.5 percent of 264 Indonesian respondents who work in health-related fields are eager to vaccinate against COVID-19 prior to the vaccine’s introduction to the country [9]. A comparable proportion was discovered in another study conducted in the Asian-Pacific area, which polled health care employees in six countries (95.6 percent of Indonesian HCWs were willing to vaccinate against COVID-19) [10].

However, there are not a lot of studies in Indonesia about COVID-19 vaccination intentions or acceptability, especially among health care workers in the country. A comprehensive examination of vaccination intention for COVID-19 is necessary to offer insights into the variables that influence the choice to become vaccinated. By analyzing the behavior and population under study, it should be possible to determine which components are most likely to be significant in developing a COVID-19 vaccination intention. The Integrated Behavioral Model (IBM), sometimes referred to as the Integrative Model, is a framework for identifying challenges on which to focus communications campaigns and behavior-change techniques [11,12,13].

IBM draws on concepts from the Theory of Reasoned Action (TRA)/Theory of Planned Behavior (TPB), the Health Belief Model (HBM), and other pertinent theories [14]. Individuals can develop a vaccination behavior if they have a strong reason to be vaccinated, are not subjected to significant environmental constraints to becoming vaccinated, understand the necessity of vaccination, and have a history of developing the same behavior [14].

Shmueli used the Health Belief Model and the Theory of Planned Behavior Model to forecast the general population’s intention to vaccinate against COVID-19. According to research, 80% of 398 respondents indicated a willingness to vaccinate against COVID-19. Male respondents, well-educated respondents, and respondents who vaccinated against influenza in the previous year all boost respondents’ propensity toward vaccinating against COVID-19 [15].

On the other hand, research that particularly describes the intention of Indonesian health care workers to vaccinate against COVID-19 has not yet been developed. As a consequence, this research was conducted to ascertain Indonesian health care workers’ willingness to vaccinate against COVID-19.

## 2. Materials and Methods

### 2.1. Theoretical Framework

The Integrated Behavioral Model incorporates aspects from the Theory of Reasoned Action (TRA), the Theory of Planned Behavior (TPB), the Health Belief Model (HBM), and Social Cognitive Theory (IBM) [11]. Using the aforementioned concepts, decades of research have shown that the most important predictor of behavior is one’s motivation or intention [11,13]. The IBM framework emphasizes intention as a critical variable, and it is composed of three components: attitude, social influence, and personal agency. The attitude construct is composed of two components. Experiential attitude refers to an individual’s emotional or affective response to the idea of engaging in the activity. The cognitive feature of instrumental attitude is that it is composed of beliefs about the desirable or undesirable outcomes or characteristics of task execution. Social influence consists of two normative components: (1) views about other people’s expectations of behavioral performance (injunctive norm) and (2) perspectives on other people’s actions with regard to that behavior (descriptive norm) [11,13]. The model used in this study is available in the Supplementary Materials (Figure S1).

To predict intention to vaccinate against COVID-19, published research generally employed HBM or TPB theories, which are the foundation of IBM constructs [6,15,16,17]. Thus far, a study utilizing IBM to predict COVID-19 vaccine intention have not yet become available.

### 2.2. Study Design

This study was conducted as a cross-sectional study from February to May 2021. The subjects of this study were all health care workers who work in Indonesia, defined based on the health care worker criteria stated in The Indonesian Government Regulation Number 36, Year 2014 (medical personnel, clinical psychology staff, nursing staff, midwives, pharmacists, public health workers, environmental health workers, nutritionists, physical therapists, medical technicians, biomedical engineers, traditional health workers, and other health workers) [18] and could access the online survey. The exclusion criteria were health care workers who were unwilling to participate in the study or who did not fill out the online survey completely.

The study was split into two parts: (1) a qualitative research-elicitation phase to identify COVID-19 vaccination concerns among a representative sample of Indonesian health care professionals and (2) a cross-sectional quantitative survey of Indonesian health care workers.

Elicitation interviews were conducted as part of the survey’s formative research. Individual qualitative interviews with fifteen HCWs were conducted in their native languages and were structured around IBM components. The participants were asked to consider receiving a COVID-19 vaccine and then to report their sentiments and views regarding the vaccine’s outcomes, sources of normative influence, and obstacles and facilitators to vaccination. The content analysis conducted for the transcribed interviews revealed lists of the participants’ sentiments, behavioral outcomes, sources of normative influence towards vaccination, and obstacles and facilitators to vaccination.

Following the content analysis, fifteen positive and negative feelings (attitude items), positive and negative beliefs, eight sources of normative influence, and seven restrictions to vaccinating against COVID-19 were discovered. The final survey questionnaire included sections on socio-demographic characteristics. To obtain the data, an online poll on an independent website in Bahasa Indonesia was employed. The study’s information was disseminated via HCW groups and social media. The participants completed an e-consent form by marking a box before taking part in the research. The consent form included a disclaimer stating that participation was voluntary and that refusing to participate had no implications. This study was conducted in accordance with the Declaration of Helsinki. The ethics committee of Universitas Indonesia’s Faculty of Medicine approved this research.

### 2.3. Survey Instrument

We developed the instrument used in this study. For all IBM constructs, the items were evaluated on a seven-point bipolar scale. Behavioral intentions were measured using three items about health care workers’ willingness to vaccinate against COVID-19: *“I hope to be able to vaccinate against COVID-19 when the schedule is available”*, *“I want to vaccinate against COVID-19 with the currently available vaccine”*, and *“I will continue to vaccinate against COVID-19 even though there are obstacles to doing so”* rated on a scale of Strongly Disagree (1) to Strongly Agree (7). The scores were averaged to provide a measure of intention: scores over 5 on a scale of 1 to 7 were classified as “intended to obtain the vaccination,” scores between 3 and 5 were classified as “unsure,” and scores below 3 were classified as “not meant to get the vaccine.”

To examine the attitudes toward conduct, the fifteen behavioral belief and experiential attitude items were multiplied by a comparable item evaluating outcome evaluation. All multiplicative scores for all items were added together. To measure the perceived norms, eight descriptive normative belief items were utilized, which were multiplied by a corresponding item assessing identification with the referent. Self-efficacy was measured using three control belief items multiplied by a perceived power item. To test perceived control, seven control belief items were employed, which were then multiplied by a perceived power item. Due to the absence of this concept during the elicitation phase, injunctive norms were not examined.

### 2.4. Data Analysis

SPSS^®^ Statistics 25 software was used to analyze the data (IBM Corp., Armonk, NY, USA). On the basis of background or sociodemographic factors, descriptive analyses were conducted. For continuous variables, the mean and standard deviation are presented. For categorical variables, percentages are provided. We used bootstrapping to account for non-normality within the linear model framework and to ensure statistical conclusion validity. The next step was to conduct analyses to identify specific beliefs underlying the IBM constructs that best explain COVID-19 vaccination intention and thus may serve as the optimal target for intervention messages. We used the enter method to run a hierarchical multiple regression on each of the IBM components and sociodemographic factors linked with intention. List of symbols used in this study is available in the Supplementary Materials (Table S2).

## 3. Results

### 3.1. Background Characteristics

**Figure 1:**
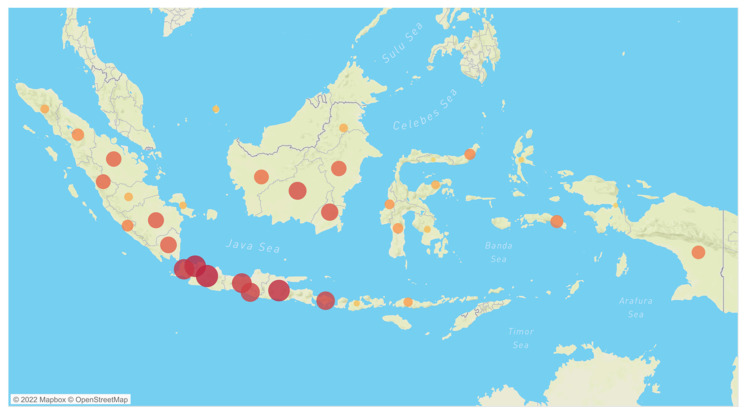
Geographic distribution of Indonesian health care workers responding to the COVID-19 vaccine intention survey (*n* = 3248).

A total of 3304 health care workers responded to the survey, of which 12 (0.36%) were removed due to insufficient data or data inconsistency and 46 (1.39%) were removed due to doubled data. Finally, data from 3248 participants were analyzed. The participating respondents represented all thirty-four provinces in Indonesia. The three most contributing provinces were located in Java: West Java (19.1%), DKI Jakarta (15.8%), and East Java (8.6%), as shown in Figure 1.

A sizable majority (61.3 percent) of respondents stated that they expect to receive a COVID-19 vaccination (scores above 5 on a scale from 1 to 7); 9.1% of participants reported that they have no intention of being vaccinated (scores below 3); and 29.6% were unsure (scores between 3 and 5). Table 1 summarizes the respondents’ demographic, socioeconomic, and other background information.

The average age of the respondents was 40.1 years, with a standard deviation (SD) of 10.6. Approximately 70% of those polled were female. More than two-thirds of respondents (77.9 percent) were married, and 23.5 percent had less than a bachelor’s degree. Surprisingly, 7.1% of respondents reported having contracted COVID-19.

### 3.2. Factor Analysis

First, we analyzed the factorability of the 15 attitude measures, 8 subjective norms items, 3 self-efficacy items, and 7 perceived control belief components. Almost all of the items had a correlation of at least 0.3 with at least one other item, indicating acceptable factorability. The communalities were all greater than 0.3, indicating that each item had some common characteristic with the others.

Because the primary objective was to identify and compute composite scores for the elements underlying COVID-19 vaccination intention, a principal components analysis with varimax rotation was performed. Cronbach’s alpha was used to determine the internal consistency of each scale. For each construct, composite scores were calculated using the mean of the items with the highest primary loadings on each factor. Table 2 contains the final items utilized in the regression. The residuals were non-normally distributed, and alternative response variable transformations had no influence on the linear regression model’s effectiveness. As a consequence, we used bootstrapping. Table 3 shows the bootstrapped (B = 1000 bootstrap samples) multiple regression results based on the given data observations.

### 3.3. Final Regression Model

The first model, which included IBM constructs (Table 3; model 1), explained 40.9% of the variance in intention to vaccinate against COVID-19 vaccine among health care workers (adjusted *R^2^* = 0.4). All constructs of the IBM model were significant predictors of COVID-19 vaccine intention (*p* < 0.01).

The final model shows that 42.5% of the variance in the intention to receive a COVID-19 vaccination was explained by the combination of both the IBM constructs and demographic variables. In this model, all constructs of the IBM model were also significant predictors of COVID-19 vaccine intention (*p* < 0.01). Furthermore, behavior belief had the most significant standardized coefficient among the IBM constructs. In other words, a one-unit increase in behavior belief increased the intention of vaccinating against COVID-19 by 0.41 units (β = 0.41, *p* < 0.01).

Five demographic characteristics were found to be strongly linked with the intent to vaccinate against COVID-19 using this model. Female health care professionals in this study showed a lower positive intention to vaccinate against COVID-19. Marital status and a personal history of COVID-19 infection were also significant negative predictors of COVID-19 vaccination intention. Furthermore, HCWs who live in the Java Island provinces and had a high income (four or more times the minimum regional income) showed a stronger favorable intention to vaccinate. The correlation of each variable is available in the Supplementary Materials (Table S1). 

The results of the multiple linear regression revealed that the constructs of experiential attitude had a significant collective effect (*F*(4, 3243) = 201.55, *p* < 0.001, *R^2^* = 0.2). The individual predictors were examined further and indicated that the phrase ‘I feel like a “guinea pig” by being the first group of people to receive a COVID-19 vaccination’ (t = −8.684, *p* < 0.001) and the item ‘The convoluted information regarding COVID-19 vaccination makes me reluctant to vaccinate’ (t = −8.627, *p* < 0.001) were the most significant predictors in the model.

According to the findings, multiple linear regression demonstrated that the dimensions of behavioral beliefs had a substantial collective influence (*F*(7, 3240) = 212.6, *p* < 0.001, *R^2^* = 0.392). The items ‘I am not worried about the side effects of COVID-19 vaccination because the benefits are greater’ (t = 13.353, *p* < 0.001) and ‘Vaccinating against COVID-19 means I contribute to herd immunity’ (t = 5.412, *p* < 0.001), and ‘As a health worker, I feel appreciated because I can be vaccinated against COVID-19 before others’ (t = 10.582, *p* < 0.001) were the most significant predictors in the model when the individual predictors were investigated further.

Additionally, combined items of perceived norms also had a significant effect (*F*(4, 3243) = 310.04, *p* < 0.001, *R^2^* = 0.327). Family members (t = 22.335, *p* = < 0.001), doctors or competent experts (t = 7.222, *p* < 0.001), religious figures (t = 3.303, *p* = < 0.001) and the President of Republic of Indonesia (t = 1.976, *p* < 0.001) were considered significant influencers of the HCWs’ intention to vaccinate against COVID-19.

Furthermore, the results of the multiple linear regression revealed that the constructs of self-efficacy had a combined significant effect (*F*(3, 3244) = 344.9, *p* < 0.001, *R^2^* = 0.273). When the individual items were investigated further, the most significant predictor in the model was ‘I have confidence that I can vaccinate against COVID-19′ (t = 25.311, *p* < 0.001). Lastly, it was found that the regression equation of only items of perceived control was not significant (*F*(4, 3243) = 1.472, *p* = 0.208, *R^2^* = 0.001).

## 4. Discussion

The purpose of this study was to determine the predictors of intention to vaccinate against COVID-19 among Indonesian health care personnel. Previous research examining vaccine attitudes indicated geographical disparities in perceptions of vaccination’s safety and efficacy. Addressing vaccine hesitancy in a particular population may be necessary due to the phenomenon’s complex features [19,20]. It is critical to understand the factors of intention to acquire COVID-19 immunization among Indonesian HCWs since these variables may vary significantly by region, culture, and socioeconomic status. Additionally, researching characteristics associated with vaccination intention is crucial for policy development and communication in the event that a vaccine becomes available. Furthermore, it aids in the strategic formulation of vaccine promotion programs that take into account the factors affecting voluntary vaccination. The IBM model used in this study was adjusted according to the results of the elicitation phase. Injunctive norms were not tested because this construct was not yielded during the elicitation phase. This might be explained by the fact that the intention to vaccinate against COVID-19 is heavily affected by external factors, instead of internal perceptions of others, due to the unfamiliarity of the disease.

The results revealed that the majority (61.3%) of Indonesian health care workers are willing to vaccinate against COVID-19, 29.6% of the population are uncertain, and 9.1% of participants do not intend to vaccinate. This finding is consistent with earlier studies. A scoping review stated that the prevalence of COVID-19 vaccination hesitancy in health care workers ranged from 4.3 to 72 percent worldwide (average = 22.51 percent across all studies with 76,471 participants) [21]. A study in France found that among 1965 respondents, only 73.1% declared themselves in favor of the COVID-19 vaccine [22]. Another research found that the range of COVID-19 vaccine intention in HCWs was between 27.6% and 76.4% [23].

Given the nature of HCWs’ jobs, one would expect them to have no issues about receiving the COVID-19 vaccination. The willingness of HCWs to vaccinate against COVID-19 serves as an important model for the entire population. However, if HCWs remain fearful of COVID-19 vaccines, it is doubtful that they will recommend them to the general public or guarantee that accessible COVID-19 vaccines are utilized in mass vaccinations. Concerns expressed by respondents regarding a hypothetical COVID-19 vaccine serve as critical targets for interventional educational activities aimed at increasing immunization rates [6,21,24]. Therefore, health promotion of COVID-19 vaccination among HCWs based on health behavior research is required.

Our study found that our proposed model, which included IBM constructs, explained 42.5% of the variance in intention to vaccinate against COVID-19 among Indonesian health care workers.

This value is higher than that of a study using a combination of HBM and TPB models, which found that the model could explain 32% of the variance in the intention to receive a COVID-19 vaccination [25]. Previous studies have found that an integrated model from HBM and TPB constructs explains from 43% to 66% of the intention to vaccinate [1,26,27,28]. Furthermore, when sociodemographic characteristics were included in the model, the IBM constructs and demographic variables explained 43.1 percent of the variance in intention to acquire a COVID-19 vaccination. All IBM model constructs were significant predictors of COVID-19 vaccine intention in this model.

Our study showed that behavior beliefs were the strongest predictors of intention to vaccinate against COVID-19. This finding is similar to earlier studies that pointed out that COVID-19 vaccination beliefs and attitude were the most significant predictors of vaccination intention [20,29]. Additionally, this study indicated that vaccination campaigns and communications emphasizing the importance of immunization for altruistic motives may be very beneficial. Two particular items used in this study, “I am not worried about the side effects of COVID-19 vaccination because the benefits are greater” and “Vaccinating against COVID-19 means I contribute to herd immunity”, were connected to this finding. Similarly, Betsch noted that individuals vaccinate if others will benefit from their vaccination [30]. This is also mentioned in the study by Chew et al. Even though our studies used different approaches, it is interesting that the act of pro-socialness, similar to the altruistic motives in our study, is highly related to vaccine acceptance in both studies [10].

Another important finding from our study is that Indonesian HCWs indeed felt more valued as a result of the government’s decision to prioritize HCWs for COVID-19 vaccination. The Indonesian government has opted to prioritize health care workers during the initial distribution of the COVID-19 vaccination [31,32] in accordance with the World Health Organization’s (WHO) demand to guarantee that COVID-19 vaccination is prioritized for the world’s health and care professionals in the first 100 days of 2021. The World Health Organization has designated 2021 as the International Year of Health and Care Workers (YHCW) [33]. The ease in obtaining the vaccine is also highlighted by the perceived self-efficacy, where most HCWs in this study agreed with the statement, “My status as a health worker makes it easier to vaccinate against COVID-19.”

Additionally, our study discovered that perceived norms had a significant effect on COVID-19 vaccination intention. Interestingly, the majority of HCWs in this survey felt that the Indonesian president expects HCWs to receive a COVID-19 vaccination, which impacts their decision to do so. This is consistent with a study conducted in China, which concluded that HCWs may view COVID-19 immunization as a work or societal obligation and that, similarly, this effect on HCWs may not be observed in nations with low levels of confidence in their government [6]. Other studies also found that respondents who trusted authorities were more likely to accept the vaccine [10,34,35]. An Asian-Pacific study on HCWs discovered many possible socioeconomic barriers such as internalized stigma, pro-socialness scale, and trust in health care authorities. This finding is in line with that of our study, where we mentioned that vaccine intention is related to government trust as part of the subjective norms construct [10].

Similarly, in research based on other prominent health behavior theories, perceived social norms can either discourage or motivate people to vaccinate, depending on the situation. Perceived social norms were the most important predictor of COVID-19 vaccination intention in a study by Kalam et al. Those who received the vaccination were more likely to report that the majority of people they knew as well as the majority of their close relatives and friends would receive the COVID-19 vaccine [36].

Previous research found that the decision to vaccinate was very much based on what their colleagues and other people who were close to them thought about the vaccine [23,37,38]. This suggests that health care facilities and institutions should promote the COVID-19 vaccination internally rather than relying on impersonal, mass-media outreach advertisements.

Consistent with previous research on vaccination intention, this study’s findings indicated that the perceived effects of vaccines, which explained HCWs’ experiential attitude toward vaccines, are a crucial factor in vaccine decision-making [21,36]. Most HCWs are concerned about the vaccine’s side effects. This implies that even in the HCW population, clear and concise educational interventions about the COVID-19 vaccine should be carried out.

A review on COVID-19 vaccine refusal on nurses also mentioned that male sex, older age, and flu vaccination history are related to COVID-19 vaccine acceptance. This finding is similar to our study, as we found that being female is related to a lower vaccination intention [39]. Moreover, a systematic review on HCW’s vaccine attitude and related factors by Li et al. also highlighted that females had higher vaccine hesitancy [40]. In the same way, previous studies have found that male HCWs were more likely to vaccinate than female HCWs [21,24,41].

Males may be more likely to accept COVID-19 vaccination because of the sex-based disparity in clinical outcomes. According to several studies, males had a higher risk of COVID-19 complications, infectivity, and death [24,42]. Furthermore, it has been hypothesized that this is owing to worries about side effects such as infertility, major side effects that render them unable to care for their family, higher sensitivity to media myths and disinformation, and dread of receiving the vaccination while pregnant [21,41]. Previous studies have suggested that tailored communication strategies are needed to increase the uptake rate of COVID-19 vaccines among HCWs [10,40].

Additionally, marital status and a personal history of COVID-19 infection were significant predictors of intention to receive the COVID-19 vaccination. Moreover, HCWs that live in provinces on Java Island and earn at least four times the minimum regional income showed a higher favorable intention to vaccinate.

These findings may have ramifications for practice as well as research. Our study of Indonesian HCWs demonstrated the critical attitudes and responses associated with numerous factors of COVID-19 vaccination intention. These findings may contribute to the development of contextualized behavioral intervention and engagement strategies for COVID-19 immunization. Along with disseminating vaccination information, health education initiatives aimed at reducing unfavorable views toward the COVID-19 vaccine among HCWs (e.g., side effects and poor efficacy) are crucial. To overcome misperceptions, clear and exact information on how vaccines are developed and tested should be widely disseminated. Additionally, the current study suggests that the IBM model may be useful in guiding future research on the factors influencing COVID-19 vaccination intention. Our study has some limitations. One of the study’s limitations might have been selection bias, since participants were required to have cellphones or laptops. Second, due to the convenience sampling utilized in this study, the majority of respondents were concentrated on Java Island despite the fact that respondents in this survey already represented all 34 provinces of Indonesia. Third, our study did not consider some of the respondents’ socioeconomic information, including employment status, type of HCW, geography, children at home, COVID infection in family and friends, and other factors that may lead to confounding variables or interactions among the variables. Additionally, our study did not calculate the content validity index as a measure of validity. However, we believe that the elicitation process and qualitative analysis can be used as a content validation process as part of the development of a behavioral intention measuring tool. Moreover, we also measured the internal consistency of each item and factor analysis for the construct validation process. Finally, vaccine hesitation may not always correspond to real vaccination behavior. It is likely that some individuals who wish to vaccinate will confront their barriers to access, while others who were originally hesitant will later be convinced and embrace vaccination.

## 5. Conclusions

This study confirms the IBM model’s robustness in predicting the intention of health-care professionals to receive a COVID-19 vaccination. Among the determining constructs, behavior belief predicted vaccination intention the best. These findings might help with the development of contextually relevant behavioral intervention and engagement strategies to increase COVID-19 vaccination, particularly among health care personnel. The results from this study can be used by policymakers and stakeholders in order to make informed decisions.

## Figures and Tables

**Table 1:** Respondent characteristics (*n* = 3248).

Variables	Estimates, *n* (%)
Sex	
Female	2274 (70)
Male	974 (30)
Age	
18–34	1105 (34)
35–55	1874 (57.7)
>55	269 (8.3)
Marital status	
Single	586 (18)
Married	2530 (77.9)
Widowed	117 (3.6)
Not willing to answer	15 (0.5)
Education	
High school/vocational high school	31 (1)
Associate’s degree	760 (23.4)
Bachelor’s degree	1079 (33.2)
Master’s degree	1270 (39.1)
Doctoral degree	108 (3.3)
Monthly income	
Up to or equal to minimum regional income (~$280)	1005 (30.9)
2–3 times more than minimum regional income	1218 (37.5)
4–5 times more than minimum regional income	419 (12.9)
>5 times more than minimum regional income	606 (18.7)
Personal history of COVID-19 infection	
Yes	229 (7.1)
No	3013 (92.8)
Not willing to answer	6 (0.2)

**Table 2:** IBM construct beliefs associated with COVID-19 vaccination intention.

Constructs and Associated Items	Corrected Item-Total Correlation	Cronbach’s Alpha (α)
*Experiential attitude*		0.754
I am concerned about the unknown long-term side effects of COVID-19 vaccination	0.439	
The convoluted information regarding COVID-19 vaccination makes me reluctant to vaccinate.	0.571	
I feel like a ‘guinea pig’ by being the first group of people to receive a COVID-19 vaccination	0.546	
I want to vaccinate against COVID-19 as long as I do not receive the vaccine that is currently available	0.431	
I am worried about the side effects after COVID-19 vaccination	0.537	
I am worried that a COVID-19 vaccination is not necessarily effective in preventing me from contracting COVID-19	0.457	
*Behavior belief (instrumental attitude)*		0.875
As a health worker, I feel appreciated because I can be vaccinated against COVID-19 before others	0.587	
A COVID-19 vaccine that has better efficacy would make me want to be vaccinated	0.369	
I am not worried about the side effects of COVID-19 vaccination because the benefits are greater	0.517	
In order for the pandemic to end, I will continue to follow health protocols after vaccinating against COVID-19	0.464	
Receiving a COVID-19 vaccination will provide peace of mind when providing services to patients	0.763	
Vaccinating against COVID-19 means I contribute to herd immunity	0.798	
COVID-19 vaccinations contribute to the end of the pandemic	0.743	
Vaccinating against COVID-19 means I provide protection for myself and my family	0.764	
Vaccinating against COVID-19 will reduce the severity of COVID-19 infections	0.652	
*Perceived norm (subjective norm)*		0.957
My co-workers expect me to vaccinate against COVID-19	0.809	
A competent doctor or expert expects me to vaccinate against COVID-19	0.835	
The president expects me to vaccinate against COVID-19	0.887	
The governor expects me to vaccinate against COVID-19	0.886	
The mayor/regent expects me to vaccinate against COVID-19	0.887	
Respected religious leaders expect me to vaccinate against COVID-19	0.843	
My immediate supervisor expects me to vaccinate against COVID-19	0.861	
My family expects me to vaccinate against COVID-19	0.704	
*Self-efficacy*		0.776
My status as a health worker makes it easier to vaccinate against COVID-19	0.541	
If I want to, it is easy for me to vaccinate against COVID-19	0.648	
I have confident that I can be vaccinated against COVID-19	0.653	
*Perceived control*		0.691
My desire to be prioritized in gaining access to health services makes it difficult for me to get vaccinated against COVID-19	0.521	
Unclear flow and procedures for gaining access to health facilities affects my decision to vaccinate against COVID-19	0.507	
My condition, which does not pass the COVID-19 screening criteria, makes it difficult for me to vaccinate against COVID-19	0.418	
Things beyond my control will make it difficult for me to vaccinate against COVID-19	0.454	

**Table 3:** Final regression model.

Variables	*B*	*SE B*	β	*R*	*R^2^*
Model 1				0.64	0.409
Behavior belief	0.007 *	2.96 × 10^−4^	0.413		
Experiential attitude	−0.002 *	3.92 × 10^−4^	−0.091		
Perceived norm	0.002 *	2.2 × 10^−4^	0.153		
Self-efficacy	0.005 *	0.001	0.145		
Perceived control	0.002 *	4.26 × 10^−4^	0.077		
Model 2				0.654	0.425
Behavior belief	0.007 *	2.98 × 10^−4^	0.412		
Experiential attitude	−0.002 *	3.92 × 10^−4^	−0.076		
Perceived norm	0.002 *	2.19 × 10^−4^	0.162		
Self-efficacy	0.005 *	0.001	0.143		
Perceived control	0.002 *	4.25 × 10^−4^	0.072		
Sex (female)	−0.161 *	0.032	−0.068		
Province (provinces in Java Island)	0.166 *	0.030	0.074		
Income (high income)	0.089 *	0.035	0.038		
Marital status (married)	−0.097 *	0.038	−0.037		
Personal history of COVID-19 infection (yes)	−0.282 *	0.056	−0.067		
Job (clinical-related jobs)	0.018	0.035	0.007		
Age	−0.003	0.002	−0.027		

* *p* < 0.01.

## Data Availability

Upon request, the authors will make the raw data accessible without restriction.

## Supplementary Materials

The following supporting information can be downloaded at https://www.mdpi.com/article/10.3390/vaccines10050719/s1, Figure S1: Integrated behavioral model. Table S1. Final regression model with additional data. Table S2. List of symbols.
