# Supplementary material for: Using Integrative Behavior Model to Predict COVID-19 Vaccination Intention among Health Care Workers in Indonesia: A Nationwide Survey
Source: Vaccines (Basel). 2022 May 4;10(5):719. doi: 10.3390/vaccines10050719 (PMC9145718; doi:10.3390/vaccines10050719)
Supplement: Supplementary file 1 [file vaccines-10-00719-s001.zip › Supplementary_Table S1.pdf]

Table S1. Final regression model with additional data.

Variables	COVID-19 vaccination intention (DV)	Behavior belief	Experiential attitude	Perceived norm	Self-efficacy	Perceived control	Sex (female)	Province (provinces in Java Island)	Income (high income)	Marital status (married)	Personal history of COVID-19 infection (yes)	Job (Clinical-related jobs)	*B*	*SE B*	β	*R*	R^2	sr^2
Model 1																0.64	0.409	0.41
Behavior belief	.599**												0.007*	2.96×10^{-4}	0.413			
Experiential attitude	-.213**	-.287**											-0.002*	3.92×10^{-4}	-0.091			
Perceived norm	.474**	.569**	-.114**										0.002*	2.2×10^{-4}	0.153			
Self-efficacy	.431**	.482**	-.071**	.446**									0.005*	0.001	0.145			
Perceived control	.109**	.037**	.308**	.145**	.153**								0.002*	4.26×10^{-4}	0.077			
Model 2																0.654	0.425	0.017
Behavior belief	.599**												0.007*	2.98×10^{-4}	0.412			
Experiential attitude	-.213**	-.287**											-0.002*	3.92×10^{-4}	-0.076			
Perceived norm	.474**	.569**	-.114**										0.002*	2.19×10^{-4}	0.162			
Self-efficacy	.431**	.482**	-.071**	.446**									0.005*	0.001	0.143			
Perceived control	.109**	.037**	.308**	.145**	.153**								0.002*	4.25×10^{-4}	0.072			
Sex (female)	-.100**	-.063**	.057**	-.027	.02	-.087**							-0.161*	0.032	-0.068			
Province (provinces in Java Island)	.064**	-.005	.006	-.035**	-.013	-.003	.044**						0.166*	0.030	0.074			
Income (high income)	.148**	.177**	-.202**	.016	.121**	-.08**	-.17**	.107**					0.089*	0.035	0.038			
Marital status (married)	.033	.117**	-.01	.068**	.068**	-.036**	-.112**	-.005	.132**				-0.097*	0.038	-0.037			
Personal history of COVID-19 infection (yes)	-.065**	.013	.049**	-.012	-.034**	.073**	-.03**	-.006	.01	.008**			-0.282*	0.056	-0.067			
Job (clinical-related jobs)	.064**	.098**	-.079**	-.013	.067**	-.023	-.012	.061**	.223**	.11**	.029**		0.018	0.035	0.007			
Age	.105**	.196**	-.097**	.105**	.079**	-0.39**	-.144**	.079**	.361**	.378**	.009	.132**	-0.003	0.002	-0.027			

*$p < 0.01$.
**$p < 0.05$.
