# Supplementary material for: Using Integrative Behavior Model to Predict COVID-19 Vaccination Intention among Health Care Workers in Indonesia: A Nationwide Survey
Source: Vaccines (Basel). 2022 May 4;10(5):719. doi: 10.3390/vaccines10050719 (PMC9145718; doi:10.3390/vaccines10050719)
Supplement: Supplementary file 1 [file vaccines-10-00719-s001.zip › Supplementary_Table S2.pdf]

Table S2. List of symbols.

Symbol	Description
p	P value
R	Coefficient of multiple correlation
R^2	Coefficient of determination
B	Unstandardized regression coefficients
SE B	Standard error for the unstandardized beta
β	Standardized regression coefficients
sr^2	Squared semipartial correlations
F	F-ratio
