# Supplementary figures and images for: Using Integrative Behavior Model to Predict COVID-19 Vaccination Intention among Health Care Workers in Indonesia: A Nationwide Survey
Source: Vaccines (Basel). 2022 May 4;10(5):719. doi: 10.3390/vaccines10050719 (PMC9145718; doi:10.3390/vaccines10050719)

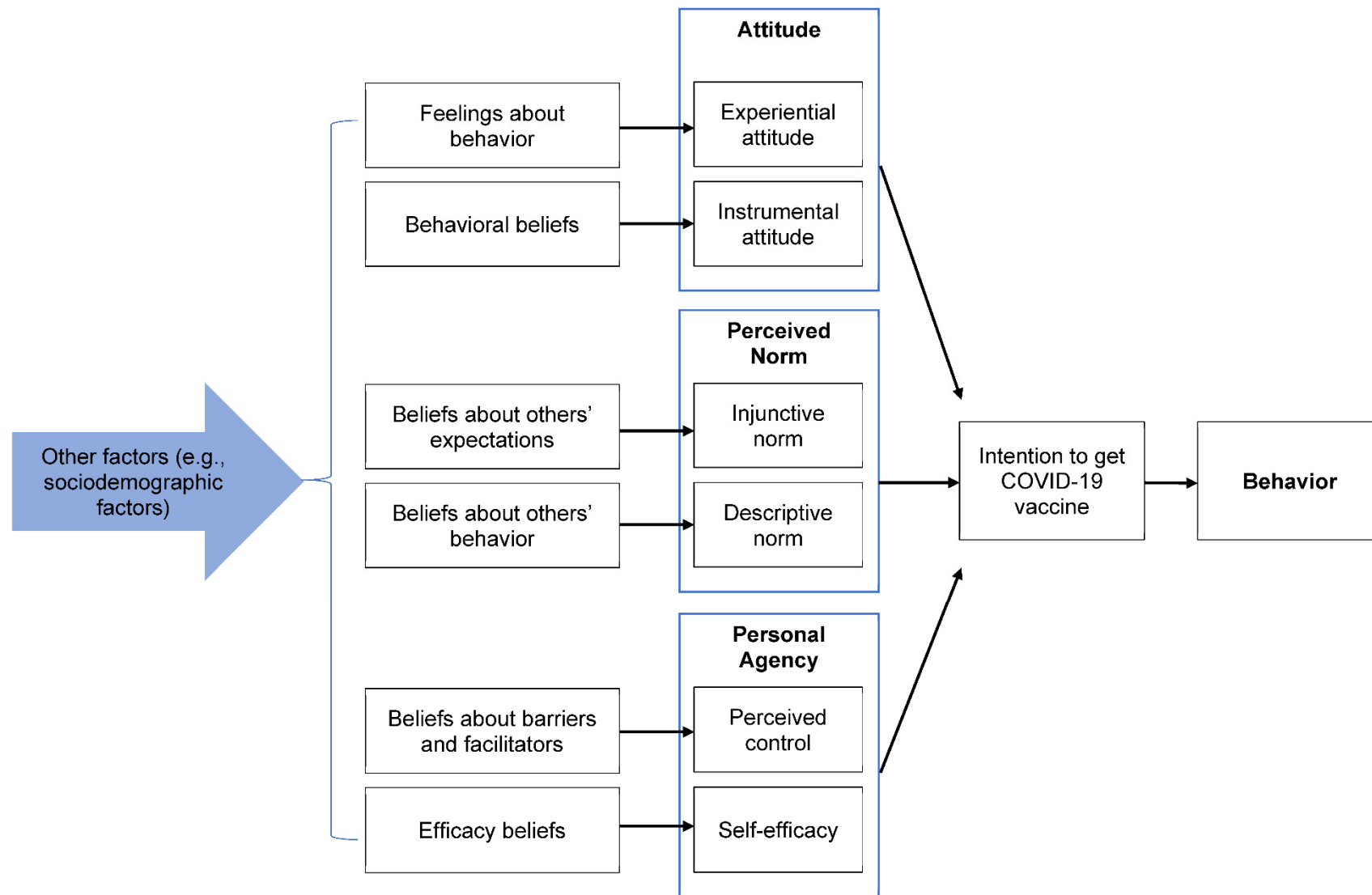

**Figure S1.** Integrated behavioral model.

Supplement: Supplementary file 1 [file vaccines-10-00719-s001.zip › Supplementary_Figure S1_IBM.pdf]
